# Bacterial Nosocomial Infections: Multidrug Resistance as a Trigger for the Development of Novel Antimicrobials

**DOI:** 10.3390/antibiotics10080942

**Published:** 2021-08-04

**Authors:** Sílvia A. Sousa, Joana R. Feliciano, Tiago Pita, Catarina F. Soeiro, Beatriz L. Mendes, Luis G. Alves, Jorge H. Leitão

**Affiliations:** 1 Department of Bioengineering, IBB-Institute for Bioengineering and Biosciences, Instituto Superior Técnico, Universidade de Lisboa, Av. Rovisco Pais, 1049-001 Lisboa, Portugal; joana.feliciano@tecnico.ulisboa.pt (J.R.F.); tiagopita@tecnico.ulisboa.pt (T.P.); catarina.soeiro25@gmail.com (C.F.S.); abeatrizmendes@tecnico.ulisboa.pt (B.L.M.); 2 Associate Laboratory i4HB—Institute for Health and Bioeconomy at Instituto Superior Técnico, Universidade de Lisboa, Av. Rovisco Pais, 1049-001 Lisboa, Portugal; 3 Centro de Química Estrutural, Instituto Superior Técnico, Universidade de Lisboa, 1049-001 Lisboa, Portugal; 4 Centro de Química Estrutural, Associação do Instituto Superior Técnico para a Investigação e Desenvolvimento, 1049-003 Lisboa, Portugal; luis.g.alves@tecnico.ulisboa.pt

**Keywords:** nosocomial infections, multidrug-resistant (MDR) bacteria, novel antimicrobial agents, drug repurposing, metal-based complexes, antimicrobial peptides, antisense antimicrobial therapeutics

## Abstract

Nosocomial bacterial infections are associated with high morbidity and mortality, posing a huge burden to healthcare systems worldwide. The ongoing COVID-19 pandemic, with the raised hospitalization of patients and the increased use of antimicrobial agents, boosted the emergence of difficult-to-treat multidrug-resistant (MDR) bacteria in hospital settings. Therefore, current available antibiotic treatments often have limited or no efficacy against nosocomial bacterial infections, and novel therapeutic approaches need to be considered. In this review, we analyze current antibacterial alternatives under investigation, focusing on metal-based complexes, antimicrobial peptides, and antisense antimicrobial therapeutics. The association of new compounds with older, commercially available antibiotics and the repurposing of existing drugs are also revised in this work.

## 1. Introduction: Bacterial Nosocomial Infections

Nosocomial infections (also known as “healthcare associated infections”—HAI) are an important cause of morbidity and mortality worldwide, being associated with a substantial increase in prolonged hospital stay and healthcare costs. According to the European Centre for Disease Prevention and Control (ECDC), a total of 8.9 million HAIs occur each year in European acute care hospitals and long-term care facilities [1]. The populations with increased risk of infections are patients in intensive care units (ICUs), surgical units, oncology/hematology units, burn units, and those undergoing organ transplant and neonates [2,3]. The most common nosocomial infections are catheter-associated urinary tract infections (CAUTI), surgical site infections (SSI), central line-associated bloodstream infections (CLABSI), ventilator-associated pneumonia (VAP), and *Clostridioides difficile* infections (CDI) [4]. Several sources of bacterial nosocomial infections have been described, including surgery procedures and invasive devices such as catheters and ventilators [4].

Several bacterial pathogens have been associated with HAI infections, with the most common HAIs being caused by *Acinetobacter baumannii*, *Burkholderia cepacia complex* (Bcc), *Pseudomonas aeruginosa*, *C. difficile*, *Clostridium sordellii*, extended-spectrum beta-lactamase (ESBL)-producing and carbapenemase-producing Enterobacterales (CPE), vancomycin-resistant Enterococci (VRE), *Staphylococcus aureus* (including methicillin-resistant *S. aureus* (MRSA), vancomycin-intermediate *S. aureus* (VISA), and vancomycin-resistant *S. aureus* (VRSA)), *Mycobacterium tuberculosis*, and nontuberculous mycobacteria (NTM). One of the major problems associated with these infections is the increased resistance of bacteria to the clinically available antibiotics. In 2019, the Center for Disease Control and Prevention (CDC) reported more than 2.8 million antibiotic-resistant infections in the United States each year, and more than 35,000 related deaths [5]. In 2019, from the above HAI common infections, the CDC included in their urgent threat list the carbapenem-resistant *Acinetobacter*, carbapenem-resistant Enterobacterales (CRE), and *C. difficile* [5]. Carbapenem-resistant *Acinetobacter* are common in ICU patients and can cause pneumonia, as well as wound, bloodstream, and urinary tract infections [6]. The control of the spread of such infections is challenging because it frequently contaminates healthcare facilities’ surfaces and shared medical equipment, causing outbreaks in these facilities [6]. CRE are one of the major concerns for patients in healthcare facilities, principally among patients requiring invasive devices (e.g., catheters) or who have long antibiotic therapy, because some bacteria of this family are resistant to nearly all antibiotics available [7]. *C. difficile* infections can cause life-threatening diarrhea and are often acquired after antibiotic treatment for other medical conditions, with the most serious infections being developed after the use of fluoroquinolones [5,8]. These infections are more common and severe in older patients [5,8].

Bacterial resistance even to one antibiotic can be a serious problem to hospitalized patients, because the use of second- and third-line treatments can have serious side effects for the patients and prolong care and recovery, sometimes for months [5]. Therefore, infection preventive measures and antibiotic stewardship are the priority in healthcare facilities to decrease the spread of antibiotic-resistant bacterial pathogens.

## 2. Antimicrobial Resistance Mechanisms of Bacterial Nosocomial Pathogens

Antimicrobials are commonly used in acute care hospitals for the treatment of both community-acquired infections and HAIs, and also for surgical prophylaxis. However, several studies have shown that this use is commonly performed using incorrect antimicrobial selection, dose, route of administration, and even the duration of the treatment [9]. This inappropriate use of antibiotics, especially broad-spectrum antibiotics, leads to the emergence and spread of antimicrobial-resistant (AMR) bacteria [10]. Another problem with unnecessary antibiotic use is the associated side effects (e.g., allergic reactions and toxicity that affects organ function) and the disruption of the human gastrointestinal microbiome, with the patient being at risk of developing problematic gut infections such as *C. difficile* [11].

Currently, the coronavirus disease 2019 (COVID-19) pandemic is boosting the use of antibiotics that could lead to the increase in AMR bacteria, because COVID-19-hospitalized patients often receive empiric broad-spectrum antibiotic therapy [12]. Besides that, hospitalization increases the risk of acquiring HAIs and also contributes to the increased use of antimicrobials [12]. Another problem is the prioritized allocation of isolation rooms to COVID-19 patients, causing cohorting and/or management in open bays of patients colonized with VRE, MRSA, CPE, or *C. difficile*, and the higher workload of healthcare workers can lead to a greater number of hospital transmissions [13].

The main cellular targets of the currently available antibiotics are the cell wall synthesis, protein synthesis, RNA polymerase, DNA replication, and folic acid metabolism [14]. The inhibition of cell wall synthesis and function, through disruption or damage of this structure, results in leakage of important solutes crucial for cellular functions, leading to cell death. The β-lactam class of antibiotics, which includes penicillins, cephalosporins, cephamycins, and carbapenems, targets the cell wall biosynthesis in bacteria. Additionally, antibiotics such as vancomycin, daptomycin, and bacitracin also interact with bacterial cell wall biosynthesis. Another mechanism of action is the inhibition of protein synthesis via irreversible binding to the 30S or 50S subunits of the ribosome. This attachment to the ribosomes results in protein synthesis interruption or in damaged proteins. As proteins are the building blocks of most cellular structures, disrupting their synthesis collapses the normal bacterial metabolism, causes death, or inhibits bacterial growth. This antibiotic class includes aminoglycosides, tetracyclines, macrolides, and streptogramins. Another antibiotic class acts by inhibition of nucleic acid synthesis by bonding to crucial components involved in DNA and RNA synthesis, and includes quinolones and rifampin. These two essential cell components are fundamental for all metabolic processes, therefore the dysregulation of the synthesis of nucleic acids will compromise bacterial multiplication and survival. Antibiotics can also be classified as antimetabolites, including sulfonamides and trimethoprim, which are responsible for the inhibition of other metabolic processes and act on selected cellular processes decisive for the survival of microorganisms.

**Figure 1:**
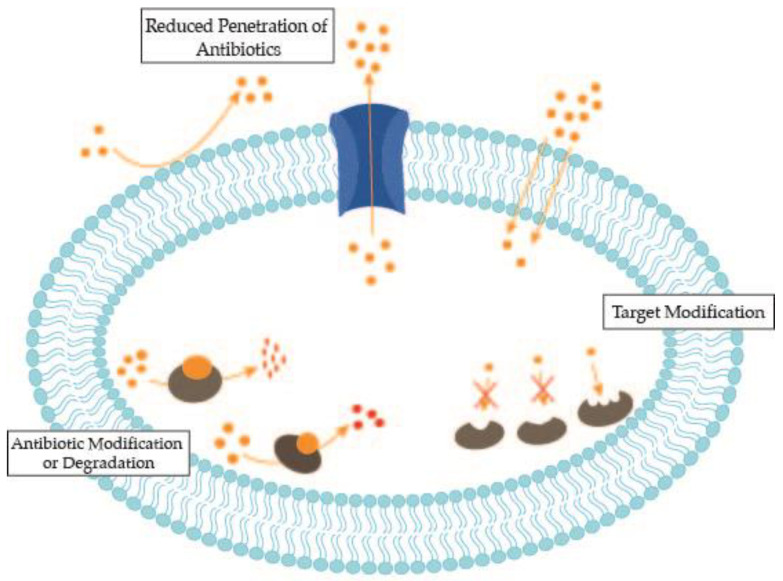
Mechanisms used by bacteria to resist antimicrobials.

However, selective pressure over bacteria leads to the emergence of resistance mechanisms, such as alterations in the outer membrane permeability, overexpression of efflux pumps, modification of the target molecule, or antibiotic inactivation [15] (Figure 1). These resistance-gene traits can be intrinsic or they can pass horizontally by mobile genetic elements [15]. The bacterial mechanisms of resistance to antibiotics have been extensively reviewed [16,17].

Due to the alarming increase of bacterial resistance rate to several antibiotics and the higher morbidity and mortality caused by these resistant bacterial strains, the Infectious Disease Society of America (IDSA) selected six priority pathogens termed ESKAPE (*Enterococcus faecium*, *S. aureus*, *Klebsiella pneumoniae*, *A. baumannii*, *P. aeruginosa*, and *Enterobacter cloacae*) as the bacterial group requiring a rapid development of new antibacterials [18].

## 3. Antimicrobial Alternatives under Investigation

**Figure 2:**
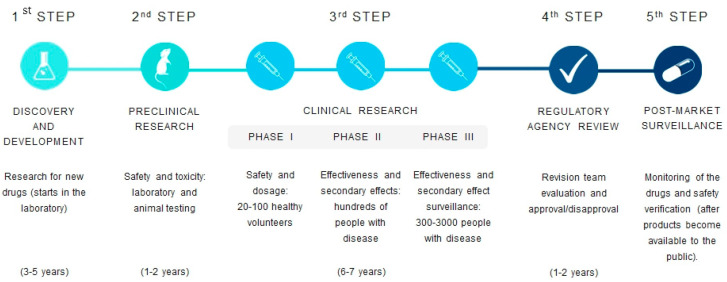
Drug discovery and development pipeline.

The development and marketing approval of new antibiotics is far behind the increasing emergence of drug-resistant bacteria. The majority of pharmaceutical companies have scaled back, or even cut, antibiotic research programs due to the increase of development challenges. In fact, only one out of five infectious disease drugs that have started testing in humans is expected to receive approval from the US Food and Drug Administration (FDA) [5]. In contrast, several research groups worldwide are focusing on the development of novel antimicrobials based on molecules with new modes of action or distinct interacting targets from those already known. The ideal antibacterial agent should be also nontoxic to the host and should have exceptional blood/fluid circulation, as well as absorption, distribution, metabolism, and excretion (ADME) properties, allowing a large therapeutic window with a low dose [14]. However, the process of drug development from a new drug discovery to commercialization is exhaustive and lengthy (Figure 2).

Despite this shortage of arrival of novel antimicrobial compounds to the market, many research groups drive their attention to the development of novel compounds and formulations to tackle the emergence of multidrug resistance. Several approaches have been developed to combat microbial infections, including the repurpose of existing drugs, the use of new delivery systems such as metal nanoparticles, the development of new compounds such as organic compounds and metal complexes, microbial metabolism-disrupting compounds such as iron and zinc chelators, antimicrobial peptides, and antisense antimicrobial therapeutics. In this review we focus on developments of the repurposing of existing drugs, novel metal-based complexes, antimicrobial peptides, and antisense antimicrobial therapeutics.

### 3.1. Repurposing of Existing Drugs

A strategy that is increasingly employed as a way of providing new drugs able to overcome the struggle of antibiotic resistance is drug repurposing. As the name implies, it consists of finding a new applicability to existing drugs, rather than their primary medical indication [19]. This approach has become especially important in an industry where the output has not been compensating the spending in the pharma resources and development and the pressure imposed by the high prices, generics’ competition, and regulatory issues, altogether inflicting a hard challenge for the discovery of new drugs [20].

Drug repurpose is rooted in two prepositions: drugs that act on various targets, and diseases that share the same biological targets [21]. Candidates to be repurposed must be drugs in clinical development or drugs safety evaluated, although having failed to show efficacy in late clinical trials. Moreover, the ones that had their project interrupted due to commercial issues are under exploitation in new geographical markets or, despite being already in the market, have generic or close to expiring patents, and thus can also be assigned to new medical indications [22]. 

The repurposing approach offers many advantages over de novo drug discovery and development. Indeed, there is a significant reduction of the processing time and development risks and costs, since repositioning candidates’ pharmacokinetic and safety profiles are already determined due to the stages of clinical development they went through [19]. In some cases, they were also submitted to other development steps, such as in vitro and in vivo screening, chemical optimization, and toxicology, which are steps that can therefore be skipped [19].

Driving the attention to MDR pathogens, there are a variety of drugs used to treat pathological conditions of noninfectious origin that have demonstrated in vitro and in vivo broad-spectrum of antimicrobial activity. Those are normally called “nonantibiotics” and can express antibacterial properties by having direct antimicrobial activity (antimicrobial nonantibiotics), enhancing the effectiveness of an antibiotic when coadministered (synergism; helper compounds), or impairing the microorganisms’ pathogenicity and activity, similar to modulating the activity of macrophages [23,24]. 

Among the existing drugs, many have proven their efficacy against MDR pathogens. Some similarities between cancer and bacterial cells have been found, such as the higher rates of replication and resistance development against chemotherapeutic agents, virulence, spread modalities within the host, and increasing aggressiveness as disease develops [25]. Thus, it is not surprising that a variety of anticancer agents, such as mitomycin C, cisplatin, and gallium, have proven their ability to fight bacteria, by crosslinking DNA or disrupting iron metabolism [26,27,28,29,30]. 

Interestingly, known for being associated with a decreased risk of sepsis, statins’ antimicrobial properties were unveiled by Jenwood and Cohen, especially against methicillin-sensitive *S. aureus* (MSSA), MRSA, vancomycin-sensitive *Enterococci* (VSE), and VRE bacteria [31]. 

The antimicrobial activity of the antihelmintic niclosamide was uncovered by Imperi et al. [32], after screening a library of FDA-approved drugs for their capacity to inhibit the quorum-sensing (QS) response of *P. aeruginosa*. Similarly, the antihistamine terfenadine was identified when searching for drugs with bactericidal activity against ESKAPE pathogens, and further confirmed by Perlmutter et al. to be active towards *S. aureus* [33]. In another study, when screening 1280 off-patent FDA-approved drugs for the inhibition of GraXRS, a two-component system of *S. aureus*, the photosensitizer verteporfin, was the most efficient in increasing polymorphonuclear (PMN)-mediated bacterial killing and reducing the bacterial load in a murine model of surgical wound infection [34]. 

Anesthetic drugs’ antimicrobial properties were first suggested in 1909, propelling further research on this topic. Aydin et al. confirmed the antimicrobial action of lidocaine and prilocaine against *P. aeruginosa*, *E. coli*, and *S. aureus.* These authors hypothesized that the antimicrobial properties of local anesthetics used for spinal or epidural anesthesia might be the reason for meningitis and other infective neurological complications being uncommon [35]. Opioids have also been applied to pain management via the epidural route, and Grimmond and Brownridge proved the antibacterial activity of pethidine against MRSA, *S. aureus*, *E. coli*, and *P. aeruginosa* [36]. 

Auranofin, an Au(I) thiolate complex, was introduced and approved by the FDA in 1985 for rheumatoid arthritis, but the toxic side effects impelled the search for less-toxic alternatives [37]. Presently, with the aim of performing drug “repurposing”, auranofin has gained increased attention, and many studies have been conducted in order to find out its mechanism of action [37]. Auranofin’s therapeutic effects have been investigated for cancer, neurodegenerative diseases, HIV/AIDS, and parasitic and bacterial infections. Harbut et al. showed that auranofin can impair the redox balance in *S. aureus* by strongly inhibiting thioredoxin reductase (TrxR) and consequently compromising their cellular defense against oxidative stress [38]. In the same study, the synergistic effect of paraquat (reactive oxygen species (ROS) generator) with auranofin was confirmed to be effective, which corroborated the damage of Trx–TrxR by auranofin and showed that this association compromises cellular defenses against oxidative stress and increases bacterial death [38]. Another interesting experiment was performed by Fuchs et al., who, after checking a higher efficacy of auranofin against Gram-positive (GSH-lacking) bacteria, tested glutathione as an antagonist of this gold complex [39]. When glutathione was introduced in auranofin Gram-positive sensitive bacteria, the MIC value of auranofin was enhanced, which reinforced the Trx system as auranofin’s target [39].

The cyclam derivative AMD3100 was found to promote a reduction of X4 HIV-1 levels in HIV-infected individuals during phase I/II clinical trials. During phase I pharmacokinetic studies, AMD3100 elevated white blood cell counts, which was later attributed to a remarkable mobilization of hematopoietic progenitor cells, in particular CD34+ stem cells, from the bone marrow into the bloodstream [40]. Meanwhile, it was found that AMD3100 is an extremely specific and effective CXCR4 antagonist. Consequently, AMD3100 was found to be efficacious in a variety of disorders that depend on the interplay of CXCR4 with its natural agonist SDF-1. These results opened the potential clinical use of AMD3100 (and its congeners) to the treatment of HIV infections as well as rheumatoid, allergic, and malignant diseases and, in principle, many other diseases that would profit from stem cell mobilization [41]. More recently, cyclam derivatives were found to be also active against a variety of Gram-positive and Gram-negative bacterial strains. Cyclams bearing substituted triazole moieties revealed a good potency against some *Mycobacterium* strains, with MIC values in the low micromolar range (3.13–6.25 μM). The *trans*-disubstituted cyclam with naphthalimide groups in the triazole moieties displays high activity against *Mycobacterium avium*, *Mycobacterium bovis*, and *M. tuberculosis* [42,43]. Replacing naphthalimide by naphthyl or benzyl groups led to a decrease in the antibacterial activity. Importantly, the inhibitory effect was maintained against clinical isolates of *M. tuberculosis* resistant to single or multiple antimycobacterial drugs, most notably strains resistant to isoniazid, rifampicin, and ethambutol. A series of *trans*-disubstituted cyclams displaying benzyl groups directly attached to the cyclam ring was also found to be active against *E. coli* and *S. aureus* [44,45]. These results revealed that the presence of a CF_3_ moiety in the benzyl groups is crucial for the antibacterial activity of the compounds. The effect of the substitution pattern revealed that changing the CF_3_ moiety from the *para* to the *meta* position in the benzyl groups led to an increase in the MIC values. The same trend was observed when a CH_2_ spacer was introduced between the CF_3_ moiety and the aromatic ring. Replacing a CF_3_ with a CH_3_ moiety led to a drastic decrease in the activity of the compound against both bacterial strains. These results suggested that the position and the polarity of the substituent on the benzyl groups attached to the cyclam ring are crucial for the antimicrobial activity of the compounds. A neamine cyclam derivative (NeaCyclam) was revealed to be highly effective against *E. coli* and *Enterobacter aerogenes* [46]. It is noteworthy that MIC values in the range of 4–16 μg/mL obtained for the resistant clinical strain *E. aerogenes* EA289 (a clinical MDR strain that overexpresses the AcrAB–TolC efflux pump) were lower than those obtained for common antibiotics belonging to β-lactams, quinolones, and phenicol families (MICs > 128 μg/mL). This compound was found to affect the outer membrane stability by altering the permeability barrier. 

Antipsychotics, antidepressants, antiplatelets, antifungals, and drugs for the treatment of multiple sclerosis are other drugs showing antimicrobial activity against bacterial pathogens. Table 1 summarizes drugs that have shown antimicrobial activity towards MDR bacteria and are, therefore, potential candidates to be repurposed as antibiotics.

It is also important to highlight that some of the drugs, besides having direct antimicrobial activity, are also able to enhance the activity of antibiotics when coadministered. This is the case of the antipsychotic penfluridol, which directly inhibited bacteria and still showed partial synergism with amikacin and gentamycin and additive effect with vancomycin and teicoplanin. This combination is expected to reduce both the antibiotic’s side effects and the occurrence of antibiotic resistance [49]. Nevertheless, there are drugs (such as the antiparasitic oxyclozanide) that, even with poor or any direct antimicrobial activity, are able to increase the antibiotics efficacy [61]. Anticancer, antidepressant, and antiparasitic agents, as well as drugs for the treatment of alcoholism and diarrhea, are also pointed out as potential helper nonantibiotics. A few examples are summarized in Table 2. 

### 3.2. Metal-Based Complexes 

Metal complexes became a fundamental pillar in medicinal chemistry after the approval of platinum in chemotherapy. Over the last two decades, metal complexes of titanium, iron, ruthenium, gallium, palladium, silver, gold, bismuth, and copper have reached clinical trials for cancer, malaria, and neurodegenerative diseases treatment [63]. The interest in metal complexes is also rising due to their ability to perform ligand exchange, generating ROS and depleting essential substrates, which is not accessible to organic compounds [63]. The shape of a molecule is one of the key factors in determining its biological fate and activity. Coordination compounds can display a vast variety of geometries and possess a more defined tridimensional arrangement in comparison to organic molecules [63]. Therefore, the structural properties of these molecules can be associated with higher clinical success rates. Lately, this research has been extended to their potential as antimicrobials, and these novel potential compounds were extensively reviewed [63,64,65]. Herein, the mode of action of silver and gold-based complexes is further described.

The antibacterial activity of silver ions was first described in the 19th century, and colloidal silver was accepted by the FDA as being effective for wound management in the 1920s [66]. However, after the introduction of penicillin in the 1940s, the use of silver diminished. In 1968, silver nitrate was combined with sulfonamide to form silver sulfadiazine, and was started to be used as a broad-spectrum antibacterial cream for the treatment of burns [67]. Silver sulfadiazine was shown to be effective against *E. coli*, *S. aureus*, *Klebsiella* sp., and *Pseudomonas* sp. It was shown that silver, but not sulfaziadine, was bound by bacteria [67]. The efficacy of silver sulfadiazine is thought to result from its slow and steady reactions with serum and other sodium chloride-containing body fluids, which allows the slow and sustained delivery of silver ions into the wound environment. More recently, clinicians started using wound dressings that incorporate silver as an alternative therapy to resistant bacteria (e.g., MRSA), because of clinical limitations of several first-line antibacterials [68]. In the last 20 years, several studies have also shown the antibacterial properties of several silver complexes against Gram-positive and Gram-negative bacteria [69,70,71]. One of these new classes are *N*-heterocyclic carbene (NHC) complexes of silver(I) that revealed interesting antimicrobial activity against resistant respiratory bacterial pathogens, including *B. cepacia* complex strains [72]. Camphor-based silver complexes have also shown promising antimicrobial properties against bacterial pathogens, being more active against Gram-negative bacteria, including *P. aeruginosa* and *Burkholderia contaminans* [70,71]. 

The exact mechanism of action of silver-based complexes on the bacterial pathogen is still not fully understood, however it has been observed to cause morphological and structural changes to bacterial cells [73]. It is suspected that the observed antibacterial effect is caused by the Ag(I) ions being released through dissociative mechanisms after entering into bacteria as coordination compounds [63]. Silver ions are proposed to react with electron donor groups (N, O, or S atoms), which are present in amino, imidazole, phosphate, carboxyl, or thiol groups in proteins and DNA [74]. Therefore, one of the possible mechanisms of action involves the interaction with thiol groups that are found in the respiratory enzymes of bacterial cells, leading to protein inactivation [74]. In the case of *E. coli*, silver ions interact with the ribosome, causing the inhibition of the expression of proteins essential for ATP production [75]. 

The antimicrobial activity of gold complexes has been shown to be intrinsically related to the ligands coordinated to the gold center, and not only dependent on the gold content [76]. For that reason, many different types of gold complexes have been synthesized and characterized, and their antimicrobial and cytotoxic activity have been assessed [77]. The main Au(I) complexes tested for antimicrobial activity contain phosphine (e.g., auranofin) or N-heterocyclic carbene (NHC) ligands [77]. The majority of the Au(I) complexes tested demonstrated selective antibacterial effects towards Gram-positive bacteria. Au(I) phosphine complexes have been gaining interest due to their easy synthesis and purification and the versatility of ligands, which provide many possibilities to rationalize the complex, according to the desired lipophilicity, hydrophilicity, and other parameters [78]. Increased interest in Au(I)–NHC complexes are due to the high stability of their coordinated compounds, great versatility of NHC ligands, and potential antimicrobial activity [79]. Some Au(I)–NHC complexes have even proved to be more efficient than silver homologues, probably due to the greater stability of the gold–NHC bond and consequent lower vulnerability to biologically active thiol groups [80]. The organic moiety was shown to have a high influence on the antimicrobial properties of the Au(I) complexes in such a way that small changes in the ligand skeleton can lead to different microbial responses [81]. Dogan et al. also observed that the antimicrobial activity might be influenced by the type of functional group bounded to the ligand [82]. Eiter et al. have shown that another important factor to consider is the charge of the complex, observing that the +1-charged complexes had better antimicrobial activity than the +2-charged complexes, which consequently had higher antimicrobial activity than +3-charged complexes [83]. However, whether these differences came from the cationic charge remains unclear, as the net charge could impair the cross-membrane transport of gold, or result from the reactivity due to the *trans*-thiourea sulfur bonding [83]. Another group of gold complexes commonly tested regarding their antimicrobial activity are the Au(III) complexes of general formula (CR)AuX_2_, in which CR is a bifunctional ligand, forming a Au–C bond, and X_2_ σ represents two unidentate anions or one bidentate anion. Together, these components form a neutral complex with a square planar geometry [84]. Just as for Au(I) complexes, Au(III) complexes antimicrobial activity is also influenced by the organic moiety. As shown by Parish et al., different ligands originated in Au(III) complexes with different antimicrobial activity, when testing AuCl_2_(damp) and AuCl_2_(ppy) complexes (being damp = 2-((dimethylamino)methyl)-phenyl and ppy = 2-pyridylphenyl) and observing that the complex with the damp ligand was more active than the complex with the ppy ligand [85]. Moreover, the antimicrobial activity was improved after synthesizing the more water-soluble complex Au(CH_3_COO)_2_(damp), especially against *S. aureus* and *E. faecalis* [86]. Reduced polarity and higher lipophilicity were also suggested to facilitate the passage of Au(III) complexes through the lipid bilayer, allowing them to inactivate diverse essential cellular enzymes, and consequently resulting in higher antimicrobial activity [87]. This was observed by comparing the antimicrobial activity of a Au(III) complex containing a quinolone ligand—a nalidix complex ([Au(nix)(Cl)_2_]) with the Ca(II), Fe(III), and Pd(II) analog complexes, standard drugs (tetracycline and amphotericin B), and the free ligand [87]. Other gold complexes that were described as displaying remarkable antibacterial activity were the tetrachloroaurate (III) complex [(H_2_thioterpy)Cl(AuCl_4_)] and the bisdithiolate Au(III) complexes [NBu_4_][Au(cdc)_2_] (where cdc = cyanodithioimidocarbonate), [PPh_4_][Au(qdt)_2_] (where qdt = quinoxaline-2,3-dithiolate), and [NEt_4_][Au(Et-thiazdt)_2_] (where thiazdt = *N*-alkyl-1,3-thiazoline-2-thione dithiolate) [88,89,90]. The bisdithiolate Au(III) complexes tested were only active against Gram-positive bacteria.

The thiol-based redox metabolism has been described as particularly vulnerable to gold complexes [38,91]. The metabolism of thiol is essential to mediate the reduction and oxidation of disulfide bonds, performing thiol–disulfide exchange between cysteines included in their active site and cysteines that belong to the substrate protein [92]. This is crucial for the proper function of regulatory processes, protein folding, DNA synthesis, and defense against oxidative stress [92,93]. The major antioxidant systems found in bacteria are Trx, glutathione (GSH), and catalase [93]. To put this in context, the thioredoxin system (Trx–TrxR) is composed of NADPH, TrxR, and Trx. This system is ubiquitous; however, some bacteria lack the glutathione system (GSH–Grx) and catalase. Most Gram-negative bacteria, such as *E. coli*, possess the three types of antioxidant systems, and the GSH system acts as a backup when the Trx–TrxR system is compromised [93]. However, in *M. tuberculosis* and many Gram-positive, and some Gram-negative, bacteria, such as *Helicobacter pylori*, the glutathione system is absent, meaning that the Trx system is entirely responsible for cellular thiol/disulfide balance and survival under oxidative stress [93]. This is the case of *S. aureus*, in which Trx–TrxR was experimentally confirmed to be essential for growth [94]. Other bacteria lack catalase, such as many streptococci, being the antioxidant function dependent on a major thiol-dependent system, which remains unclear [93]. 

Other researchers have found that gold complexes (e.g., auranofin) can cause the inhibition of multiple biosynthetic pathways in *S. aureus* [57]. When tested in subinhibitory concentrations, auranofin inhibited cell wall and DNA synthesis (although not by intercalation), and when in higher concentrations, auranofin inhibited protein synthesis. The downregulation of five major biosynthetic pathways (lipid, cell wall, protein, RNA, and DNA), and the inhibition of the production of the major *S. aureus* toxins, Panton–Valentine leucocidin (PVL) and α-hemolysin (Hla), were observed upon exposure to auranofin [57].

However, some resistance to gold complexes was observed in some bacterial pathogens. One of the mechanisms underlying resistance was the reduced uptake caused by membrane reduced permeability. This finding was reported by Marzo et al., who proved increased susceptibility when administering auranofin together with a permeabilizing agent, such as polymyxin B [95]. These authors showed that the lack of antibacterial activity of auranofin was due to the lower outer membrane permeability instead of glutathione alone [95]. The presence of efflux pumps was also suggested to be part of the resistance mechanisms of Gram-negative against auranofin, which was confirmed by Thangamani et al. after deleting the efflux pump AcrAB in *E. coli* and observing a four-fold decrease in the auranofin’s minimum inhibitory concentration [57]. Liu et al. compared the antibacterial activity of Au(III) complexes with their Au(I) analogues and observed that the Au(III) complexes were more active against Gram-positive than the Au(I) analogue [96]. Both had low activity against Gram-negative bacteria.

Trimetallic complexes with {Pt_2_Au(µ-S)_2_}^n+^ (n = 2, 3) cores containing C, N and N, N donor ligands were also synthesized and their antimicrobial activity was assessed by White et al. [97]. However, these complexes exhibited a reduced antimicrobial activity when compared to other cycloaurated gold(III) complexes.

### 3.3. Antimicrobial Peptides 

Antimicrobial peptides (AMPs), a diverse group of bioactive small proteins, are essential host defense components that are ribosomally synthesized by most lifeforms, including bacteria, archaea, fungi, plants, and animals [98,99]. Natural AMPs are usually rich in positively charged amino acid residues (such as lysine, arginine, and histidine), have an amphipathic nature, and show a broad spectrum of activity against a wide range of pathogens (bacteria, fungi, parasites, virus) [100,101]. Structurally, these peptides are generally divided into four categories (linear α-helical, β-sheet, extended, and cyclic peptides), and their physicochemical properties influence the action mechanism of AMPs. The stereotypical mechanism of AMP action is to integrate into the bacterial cell membrane and disrupt its integrity, leading directly or indirectly to bacterial cell lysis and death [102,103]. Although bacterial killing by AMPs commonly occurs via membrane perturbation mechanisms, they can also exhibit more complex activities, including bacterial cell penetration and interference with vital intracellular processes (e.g., metabolic and translation inhibition) [104,105,106].

In addition to the fast killing kinetics, pharmacodynamic properties, and mechanisms of killing that overcome common resistance mechanisms of MDR pathogens, AMPs may elicit an anti-infective host immune response and possess the ability to neutralize toxins [107,108,109,110]. The antibiofilm properties of these molecules may also confer efficacy against infections associated with wounds, medical implants, and chronic respiratory illnesses [111,112,113]. 

Considering the critical status of bacterial resistance, several attempts have been made to find AMP-based effective therapeutics. To date, there are numerous clinically relevant AMPs that were reported to show antimicrobial, antibiofilm, anti-inflammatory, and/or wound healing abilities. However, only a few of these peptides have proceeded to preclinical studies or clinical trials, obtained FDA approval, or have been launched on the market. 

Due to their antimicrobial potency, nisin, gramicidin, polymyxins, daptomycin, and melittin were approved for clinical use. Nisin, a polycyclic antibacterial peptide naturally produced by lactic acid bacteria, is an FDA-approved and generally regarded as safe (GRAS) peptide that is mostly used in Europe as an antibacterial food additive [114,115]. However, the safety profile, together with the broad-spectrum bactericidal activity, including drug-resistant bacterial strains such as MRSA, *Streptococcus pneumoniae*, Enterococci, and *C. difficile*, indicated that the application of nisin could extend beyond food-related bacteria [115,116]. Melittin, a small peptide with anti-inflammatory properties, and the main component of the venom of the honeybee *Apis mellifera*, was approved by the FDA for relieving pain and swelling associated with some inflammatory diseases [117,118]. Similar to nisin, the action of melittin against drug-resistant bacteria in several in vitro and animal experiments suggests that the clinical application of this peptide could be extended beyond the FDA-approved purposes [119,120]. Gramicidin and daptomycin are effective against a variety of Gram-positive bacteria, including antibiotic-resistant strains [121,122]. Daptomycin is intravenously administrated and can be used in combination with β-lactam, improving the clinical outcomes in patients with MSRA bloodstream infections [123]. Despite its bactericidal, antipersister, and antibiofilm activities towards various relevant clinical strains, gramicidin is only used as a topical agent, especially for ophthalmological purposes, due to its cytotoxicity [124]. Polymyxins are a group of naturally occurring cyclic polypeptides that show activity against MDR Gram-negative bacteria, such as *P. aeruginosa* and *E. coli*, their main target being the lipopolysaccharide [125]. Polymyxin B, usually prescribed to treat eye infections, and polymyxin E (colistin), used to treat wound infections, are crucial and in clinical use; however, due to their severe toxicity, they are reserved as last-resort treatment options [126,127]. Polymyxins consist of a cyclic heptapeptide core, which is linked to a pendant acyl long chain bridging through an exocyclic linear tripeptide moiety, and these are important structural features required for showing antibacterial activity. Additionally, positive charge provided by the cationic amino acid residue di-amino butyric acid and a significant hydrophobicity due to the amino acids phenylalanine and leucine play an essential role in their effectiveness. Cyclam-based derivatives have been designed to mimic this class of antibiotics [128]. The cyclic molecular backbone was mimicked by the incorporation of cyclam, and the lipophilicity was promoted by conjugating different long-chain fatty acids. Phenylalanine and leucine were also introduced in the structure. The results revealed that longer aliphatic chains led to an increase in the antibacterial activity against *A. baumannii*, *P. aeruginosa*, *E. coli,* and *K. pneumoniae* [128]. The most active compound showed a potent activity in the concentration range of 2–8 μg/mL to both wild-type and drug-resistant clinical isolates of those bacteria. The compound was found to be also highly active against MRSA, with a MIC value of 2 μg/mL. The ex vivo antibacterial activity against *P. aeruginosa* ATCC 27853 and *P. aeruginosa* L-2026/17 portrayed its effectivity in the treatment of a human corneal infection model by reducing the bacterial burden by 1.5 log for *P. aeruginosa* ATCC 27853 and by 1.9 log for the drug-resistant clinical isolate obtained from an ocular infected eye [128].

Based on promising preclinical results showing successful broad-spectrum bacterial activities in in vitro and in vivo models, numerous AMPs have been investigated in human clinical trials, but the low number of AMPs approved for clinical use is discouraging. Omiganan, pexiganan, surotomycin, and Neuprex, which have completed advanced clinical trials, failed in phase III because they did not show the expected effectiveness [129,130]. Other trials were not successful because of the increased mortality in treatment groups versus control group (such as in the case of talactoferrin [131]) or the increased toxicity observed in the treated group. Murepavadin is an example of a cyclic peptide specifically potent against *P. aeruginosa*-associated nosocomial pneumonia, whose phase III clinical trial was prematurely ended due to acute renal toxicity in the treated group [132].

Amongst the challenges that hamper the in vivo efficacy of AMPs and delay their successful development for clinical use are (A) the potential loss of activity in the presence of low pH, saline, divalent cations, and serum or plasma proteins; (B) liability to degradation by tissue proteases; (C) potential hemolytic and/or cytotoxic effects; (D) potential immunogenicity and unclear pharmacokinetic properties; (E) low antimicrobial activity in clinically relevant environments, which is particularly important in the context of sepsis as a complication of MDR-associated wound infections; and (F) potential resistance to AMPs, although far less common than resistance to current antibiotics [133,134]. Several strategies have been developed to overcome the mentioned limitations and reduce the production costs of AMPs. Ultra-short and/or truncated AMPs, such as LTX-109, a synthetic tripeptide with low propensity for resistance development that can prevent infections by MSSA/MRSA during hospitalization [135], have been pursued by several companies to reduce the production costs. 

The development of novel delivery approaches to administer the peptides and the introduction of chemical modifications have been also used to improve their bioavailability and efficacy in vivo. Strategies in the design of modern AMP–polymer surfaces have been optimized, and different nanocarriers (such as novel polymeric and lipidic nanoparticles, carbon nanotubes, micelles, liposomes and cubosomes, polymersomes, microspheres, dendrimers, nanocapsules, and other colloidal delivery systems) have been loaded with AMPs, facilitating the transport and the delivery of these peptides, and offering added value in smart biomedical applications [136]. For instance, to reach the colon and target *C. difficile*, nisin was encapsulated in pectin/ hydroxypropylmethyl cellulose (HPMC) compression-coated tablets, forming an enzymatically controlled delivery system [137]. *E. coli* and *S. aureus* bacterial adhesion was inhibited using the Magainin II peptide covalently immobilized over poly(lactide-co-glycolide) (PLGA) and electrospun PLGA/gelatin fibers [138]. Compared with plain surfactin, gold nanodots comobilized with the AMP surfactin demonstrated a superior antimicrobial activity not only against non-MDR and MDR (including MRSA) bacteria in vitro, but also showed a faster healing and better epithelialization on MRSA-infected wounds in vivo in rats [139]. There is also a wide range of chemical modifications that can be used to improve the stability of AMPs, including the conjugation of different AMPs, the development of synthetic mimics of antimicrobial peptides (SMAMPs), and the use of peptoids, a class of peptidomimetics consisting of N-substituted glycine oligomers. The cationic peptide SA4 and its poly-n-substituted glycin homolog SPO are an example of peptoids that inhibit the planktonic and biofilm formation of *A. baumannii* strains, which are recognized to be among the most difficult to control and treat antimicrobial-resistant Gram-negative bacilli [140]. It was also reported that the hybrid peptide PA2–GNU7, constructed by the addition of PA2 to GNU7, has a high activity and specificity to *P. aeruginosa* [141]. Interestingly, the chimeric peptides KG18 and VR18, conjugated with tungsten disulfide quantum dots, also showed antibacterial and antibiofilm activity against *P. aeruginosa* [142]. 

Combination therapies have also been explored to improve the clinical outcomes of antibacterial treatments. The synergism between AMPs and conventional antibiotics, or among different AMPs, have also been investigated, and some favorable results have been documented. For instance, the combination of polymyxins with carbapenems or rifampicin suppresses the development of polymyxin resistance [143]. In vitro data suggested that the combination of colistin with anthelmintic salicylanilides could be an effective killing strategy against *A. baumannii*, *K. pneumoniae*, *P. aeruginosa,* and *E. coli* MDR clinical isolates [61]. In addition, the synergistic effect of the proline-rich antibacterial peptide A3-APO with colistin was also reported both in in vitro assays and in a *K. pneumoniae*-infected bacteremia mice model [144]. It seems that the combination therapy may not only improve the efficacy of AMPs, but also reduce their cytotoxic effects and reduce the treatment costs [129]. 

In summary, AMPs offer a hopeful alternative to conventional therapeutics; however, a thorough understanding of their structure and interaction with bacterial and host cells is still needed to develop a safe, stable, and efficient antimicrobial product. 

### 3.4. Antisense Antimicrobial Therapeutics 

Antisense RNAs (asRNAs) are ubiquitous in bacteria and are involved in a wide range of functions, from central metabolism to pathogenesis-related mechanisms [145,146]. This strategy can be turned to our favor by using synthetic asRNAs to fight pathogens, targeting metabolism and/or antibiotic resistance genes [147]. Antisense therapies are emerging as one of the best alternative strategies over classic antibiotics, promising to greatly reduce the time required to discover new antimicrobials and enabling therapies specific to a target gene and microorganism. However, these therapies are still far from being a common antimicrobial approach, mostly due to the challenge of delivering oligomers to bacterial cells [148]. 

Antisense oligomers (ASOs) act by binding to target mRNAs with a complementary sequence. This interaction inhibits the mRNA translation into protein through steric blockage and/or through RNase degradation of the ASO/RNA duplex [149]. The first step to make this therapy clinically possible is to chemically modify the ASOs. The modification of ASOs sugar, backbone, nucleobase, and 3′- and 5′-terminal can improve their stability, avoid nucleases attacks, and preserve target specificity [150]. ASOs modifications are mainly four: phosphorothioates (PS) linkages, locked (bridged) nucleic acids (LNA/BNA), peptide nucleic acids (PNA), and phosphorodiamidate morpholino oligomers (PMO) [148]. ASOs modifications have been recently reviewed by Hegarty and Stewart [151]. To address the impossibility of delivery-free antisense oligomers (ASO) through the cell wall, some strategies using carriers have been developed. Cell-penetrating peptides (CPPs) and diverse nanomaterials are currently the most studied delivery systems to introduce ASOs inside bacteria [151].

Wesolowski et al. developed a PMO conjugated with CPP to target a highly conserved region in the *E. coli gyrA* gene sequence [152]. The authors have shown that targeting *gyrA* with a CPP–PNA reduces the viability of some pathogens such as *E. faecalis*, *S. aureus*, *P. aeruginosa*, and *S. pneumoniae*, as well as of *Streptococcus pyogenes*. While the CPP used has a bacteriostatic effect, the PMO is bactericidal, and this combination leads to an enhanced microbial effect [152]. In addition, targeting the *S. pyogenes gyrA* can have a synergistic effect when applied alongside levofloxacin, novobiocin, or spectinomycin [153]. Barkowsky et al. tested several CPPs and observed that HIV-1 TAT, Oligolysine (K8), and (RXR)4XB were the most efficient to abolish bacterial growth in vitro [154].

Równicki et al. were able to activate the *mazEF* and *hipBA* toxin–antitoxin system of *E. coli* using a PNA anti-*hipBA*, observing growth arrest [155]. In another study, with PNAs targeting the *ftsZ* gene in *S. aureus*, required for cell division, an inhibited growth and decreased gene expression were also observed [156]. 

The use of ASOs for targeting antibiotic resistance genes is also being widely explored. Oh et al. showed a restored susceptibility to ciprofloxacin and erythromycin by targeting the multidrug efflux pump genes *CmeABC* of *Campylobacter jejuni* with PNAs [157,158]. Wang et al. have shown that a PNA targeting the mobilized colistin resistance (*mcr-1*) gene restored *E. coli* colistin susceptibility [159]. In another work, targeting the outer membrane protein OprM gene of *P. aeruginosa* with a phosphorothioate oligodeoxynucleotide led to a reduced resistance to multiple antibiotics [160]. It is noteworthy that in this work the authors used, for the first time, anionic liposomes to encapsulate the ASO to promote their internalization. Recently, Al Husseini et al. focused on *P. aeruginosa* persisters, highly drug-tolerant cells, and were able to eradicate in vitro *P. aeruginosa* persisters using a PNA anti-*mqsR* [161]. 

Encapsulation within liposomes and conjugation with lipids are still being explored as delivery systems. Some of the latest developments include the use of cationic fusogenic liposomes and lipid oligonucleotides to improve the internalization efficacy [162,163]. Kauss et al. reported an effective reduction of the resistance level to ceftriaxone in *E. coli* cells harboring blaCTX-M-15 by using an oligonucleotide conjugated to a lipid moiety [163].

Bioinformatics is also bringing a boost for antisense therapies, with the facile accelerated specific therapeutic (FAST) platform being the most recent platform to develop antisense therapies to combat MDR bacteria [164]. Aunins et al. used FAST to create PNAs against CRE bacterial genes identified by transcriptomics. These authors observed a potentiated carbapenem efficacy by targeting *hycA*, *dsrB*, and *bolA* in CRE *E. coli*, whereas targeting *flhC* and *ygaC* conferred added resistance [165]. The use of transcriptomics with highly efficient bioinformatics platforms can be a game changer in fighting MDR bacteria.

Using a mouse model of infection, Geller et al. were pioneers in showing the efficacy of a PMO (at the time without any vehicle) in an animal model. Targeting the essential *acpP* gene of *E. coli*, a reduced viability both in culture and in the peritoneum of the infected mice was observed [166]. Recently, Hansen et al. have shown the efficacy of a PNA anti-*acpP* against colistin- and tigecycline-resistant *E. coli* and *K. pneumoniae*, whereas Castillo et al. reported a synergistic effect of anti-*acpP* with trimethoprim and polymyxin B against pathogenic *E. coli* [167,168]. Castillo et al. used the CPP (KFF)3K that was shown by others to be crucial for the PNA to cross through the LPS/outer membrane as well as the inner membrane [167,169]. The Greenberg Lab group also assessed the effect of a CPP–PMO to target the antibiotic-resistance gene New Delhi metallo-β-lactamase (NDM-1), resulting in an improved survival (92%) and reduced systemic bacterial burden when administered concomitantly with meropenem to *E. coli* CVB-1-infected mice [170]. The same research group also targeted the *acpP* gene of *Acinetobacter lwoffii* and *A. baumannii*. These authors treated a murine pulmonary infection model with an intranasal (RXR)4-AcpP treatment that led to a survival rate of 100% [171]. Targeting *A. baumannii aac(6′)-Ib*, a gene important for resistance to aminoglycosides, increased the susceptibility to amikacin, an effect already described for *E. coli* [172,173]. An increased survival rate of infected *Galleria mellonella* with a synergistic treatment was observed [172]. This additive phenomenon was also recently described when targeting *lpxB*, a gene essential for maintaining the structure of the bacterial cell envelope, combined with colistin treatment [174]. Those works are paving the way for a new viable therapeutic approach in dealing with MDR *Acinetobacter* species. 

LNAs targeting *aac(6′)-Ib* conjugated with different CPPs have shown a wide range of inhibitory effects towards *K. pneumoniae* JHCK1, *A. baumannii* A155, and *E. coli* TOP10 (pNW1), evidencing the particularity of each organism [175,176,177]. Targeting *acpP* was also tested for *P. aeruginosa,* similar to the mentioned Geller et al. experiment with *Acinetobacter*, but Howard et al. also targeted other essential genes such as *lpxC* and *rpsJ*. Although the target genes are not directly related to antibiotic resistance, an inhibitory effect was observed in vitro and in vivo, which can also be attributed to synergy with antibiotics [178]. Targeting *acpP* was also described to inhibit bacterial-induced inflammatory responses in CF IB3-1 cells infected by *P. aeruginosa* PAO1 [179]. An LNA anti-*acpP* encapsulated in niosomes has also shown some promising results for *P. aeruginosa* [180]. CPP–PMOs targeting *acpP* have also shown interesting results for another group of opportunistic lung pathogens, the Bcc group, where a reduced pathogenicity was observed in CGD mice infected with *Burkholderia multivorans* [181]. Sawyer et al. showed that a CPP–PMO targeting *gyrA* (an essential gene required for replication) was able to reduce the viability of *S. aureus* in a mouse cutaneous wound infection with a topical application delivery system [182]. Meng et al. also reported the efficacy of a CPP–LNA against *S. aureus*. Targeting *ftsZ*, which is required for cell division, these authors observed an inhibitory effect in vitro as well as in infected mice, showing a reduced level of *ftsZ* mRNA and FtsZ protein expression and an increased survival of mice by 60% [183]. PNAs targeting *mecA* and *ftsZ* have been shown to increase susceptibility to oxacillin of MRSA and *Staphylococcus pseudintermedius* [184]. The use of a PS to target *mecA* in combination with oxacillin has also been proven to improve survival rate by 30–50% in *S. aureus*-infected mice [185]. RNAP primary σ^70^ have also been suggested as a good target for developing novel antisense antibiotic to treat severe MRSA infections [186]. 

Intracellullar pathogens can be even more challenging for antisense therapies since there is an extra membrane to pass through. One of those pathogens is *Listeria monocytogenes*. Abushahba et al. tested five CPP–PNA conjugates targeting the *L. monocytogenes* RNA polymerase α subunit (*rpoA*). The designed conjugates were capable of silencing *rpoA* and killing the bacteria in pure culture, in infected macrophage cells, as well as in a *Caenorhabditis elegans* animal model, with (RXR)4XB being the most effective vehicle [187]. Rajasekaran et al. also used a CPP–PNA to target some genes of *Brucella suis*, a facultative intracellular pathogen responsible for brucellosis. Targeting *polA*, a DNA polymerase necessary for DNA replication, was the most effective PNA in broth culture, where the PNAs targeting *asd* and *dnaG* revealed the higher inhibitory effect in *B. suis* infecting macrophages [188]. These differences of effectiveness of PNAs from in vitro to in vivo assays highlight the differences of bacteria metabolism in different environments, as well as the importance of using infection models to test the efficacy of this kind of therapeutics.

## 4. Conclusions

Antibiotic resistance emergence in bacterial pathogens is a global threat to public health systems, not only by the eminent inefficacy of antibiotics against these resistant microorganisms, but also by the associated increase of the risk of performing invasive and noninvasive medical treatments and procedures that rely on antibiotics administration to reduce complications. 

The screening of reservoirs of existing nonantibiotic drugs is leading to the identification of new antibacterials, having the advantage of reducing the necessary time for the antibacterial drugs to be approved and available in the market. However, drug repurposing will not solve all nosocomial bacterial infections and drug resistance-associated problems. Therefore, novel compounds with antibacterial activity are also required to tackle these difficult-to-treat infections. These new antimicrobials could lead to the identification of new bacterial targets. 

An overview on the efforts by several research groups to develop novel antimicrobials evidences a wide diversity of molecules and approaches in use, expected to lead, in the near future, to their introduction in the market. These novel and effective antimicrobials are urgently needed to combat nosocomial infections.

However, the mechanisms of action of the majority of these new compounds are not clear. Omics technologies could be a helpful strategy for the identification of their drug targets and the bacterial mechanisms of resistance [189].

## Figures and Tables

**Table 1:** “Nonantibiotic” approved drugs with antimicrobial activity towards MDR bacteria.

Drug	Primary Medical Indication	Antimicrobial Activity	Mechanism of Action	Reference
Mitomycin C	Anticancer	*E. coli, S. aureus, P. aeruginosa*	Crosslinking DNA. Eradicates cells in biofilms.	[26]
Cisplatin	Anticancer	*E. coli, S. aureus, P. aeruginosa*	Crosslinking DNA. Effective against biofilm and planktonic cells of *P. aeruginosa*.	[27]
Gallium	Anticancer	*P. aeruginosa* (CF patients isolates), *K. pneumoniae, A. baumannii*	*P. aeruginosa*: Disrupts iron metabolism and increases oxidant sensitivity. *K. pneumoniae*: reduces CFUs and biofilm. *A. baumannii:* disrupts the iron metabolism.	[28,29,30]
5-fluoro-2′-deoxyuridine	Anticancer	MRSA, VRSA, MSSA, VISA, *E. faecium; Enterecoccus faecalis*	Inhibits thymidylate synthase and impairs nucleic acids’ metabolism and structure.	[47]
Curcumin	Anticancer	*P. aeruginosa, S. aureus*	Causes nucleic acid and protein leakage, indicating impairment of the membrane.	[48]
Simvastatin	Statin	MSSA, MRSA, VRE, VSE	Targets HMG-CoA reductase in eukaryotes. However, no mechanism described for prokaryotes.	[31]
Niclosamide	Anthelmintic	*P. aeruginosa*	Inhibits the QS response and the production of acyl-homoserine lactone signal molecules. Inhibits the transcription of genes related to adhesion and biofilm formation.	[32]
Terfenadine	Antihistamine	*S. aureus, E. faecium, E. faecalis*	Type II topoisomerase inhibitor. Targets both DNA gyrase and topoisomerase IV. Antimicrobial activity against planktonic, biofilm and small-colony variant (scv) forms of *S. aureus*.	[33]
Verteporfin	Photosensitizer	*S. aureus*	Inhibits the GraXRS-dependent promoter. Sensitizes bacteria against PMN cells.	[34]
Lidocaine	Anesthetic	*P. aeruginosa, E. coli, S. aureus*	No mechanism described.	[35]
Prilocaine	Anesthetic	*P. aeruginosa, E. coli, S. aureus*	No mechanism described.	[35]
Bupivacaine	Anesthetic	MRSA, *S. aureus, E. coli*	No mechanism described.	[36]
Pethidine	Opioid	MRSA, *S. aureus, E. coli, P. aeruginosa*	No mechanism described.	[36]
Penfluridol	Antipsychotic	*E. faecalis*	Eradicates biofilm, probably inhibiting the QS system and the second messenger c-di-GMP.	[49]
Thioridazine	Antipsychotic	MRSA, *Enterococcus* species	MRSA: Inhibits bacterial efflux pumps. Inhibits replication of phagocytosed MRSA or causing ultrastructural changes in the cell envelope and consequent lysis after phagocytosis.	[50]
Sertraline	Antidepressant	*S. aureus, E. coli, P. aeruginosa*	Hypothesized to inhibit efflux pumps in bacteria once it is a serotonin reuptake inhibitor in humans. However, further studies are required.	[51]
Ebselen	Anti-inflammatory, antioxidative and antiatherosclerotic (safety proven, but not clinically used)	MRSA, VRSA, MSSA, VISA, *E. coli*, *E. faecium, E. fecalis*	Inhibits the TrxR system.	[47,52]
Ibuprofen	Anti-inflammatory	*P. aeruginosa, Burkholderia* species, *E. faecalis*	Suggested to uncouple oxidative phosphorylation in bacteria, to alter bacterial hydrophobicity, hemolysin production and inhibit fimbriae. Reduces biofilm biomass.	[53,54]
Diclofenac	Anti-inflammatory	*E. faecalis*	Suggested inhibition of bacterial DNA synthesis or impairment of membrane mechanisms. However, further studies are required.	[54]
Ticagrelor	Antiplatelet	MSSA, GISA, MRSA, VRE	Inhibits MRSA and VRE biofilm formation.	[55]
Auranofin	Antirheumatic	MRSA, *E. faecium, E. faecalis*	MRSA: Downregulates the proteins of 5 major biosynthetic pathways (DNA, RNA, protein, cell-wall, and lipid synthesis). Reduces MRSA toxins. Eradicates intracellular MRSA in infected macrophages. *Enterococcus*: Antibiofilm activity. Inhibits selenium metabolism and selenoenzymes.	[56,57]
Glatiramer acetate	Multiple sclerosis	*E. coli*, *A. baumannii, P. aeruginosa* (CF patients isolates)	Forms intracellular condensates.	[58]
Ciclopirox	Antifungal	*P. aeruginosa, Proteus mirabilis, E. coli, K. pneumoniae, S. aureus, Corynebacterium* spp., *A. baumannii*	Affects LPS composition and galactose metabolism in *E. coli*.	[59,60]

CF: cystic fibrosis; GISA: glycopeptide intermediate *S. aureus.*

**Table 2:** “Nonantibiotic” approved drugs that synergize with antibiotics towards MDR bacteria.

Drug	Primary Medical Indication	Synergy with	Antimicrobial Activity	Reference
Gallium	Anticancer	Colistin	*A. baumannii*	[30]
Curcumin	Anticancer	Ciprofloxacin	*P. aeruginosa; S. aureus*	[48]
Penfluridol	Antipsychotic	Amikacin, GentamycinAdditive effect: Vancomycin, Teicoplanin	*E. faecalis*	[49]
Thioridazine	Antipsychotic	Vancomycin, Ampicillin	*Enterococcus* species	[50]
Sertraline	Antidepressant	Ciprofloxacin, Norfloxacin, Moxifloxacin, Gentamicin, Levofloxacin. Resistance of *E. coli* to cefexime was reversed.	*S. aureus, E. coli, P. aeruginosa*	[51]
Salicylanilides (Oxyclozanide, Rafoxanide, Closantel)	Internal antiparasitic for veterinary use	Colistin	*E. cloacae, A. baumannii, K. pneumoniae, E. coli, P. aeruginosa,*	[61]
Disulfiram	Alcoholism treatment	Minocycline	*S. aureus*	[62]
Loperamide	Diarrhea treatment	Minocycline	*P. aeruginosa; E. coli*	[62]
Ticagrelor	Antiplatelet	Rifampicin, Ciprofloxacin and Vancomycin	MRSA	[55]
